# Functional Characterization of the Histidine Kinase BaeS Reveals Critical Residues for BaeSR-Dependent Stress Signaling in *Escherichia coli*

**DOI:** 10.3390/microorganisms14051031

**Published:** 2026-05-01

**Authors:** Shurong Chen, Zhengfei Qi, Lina Wang, Lian Wu, Jiayi Xie, Rui Ma, Kexin Zhang, Tong Ji, Min Zhou, Lingli Zheng, Qingshan Bill Fu

**Affiliations:** 1 Shanghai Institute of Materia Medica, Chinese Academy of Sciences, Shanghai 201203, China; chenshurong@simm.ac.cn (S.C.);; 2 Zhongshan Institute for Drug Discovery, Shanghai Institute of Materia Medica, Chinese Academy of Sciences, Zhongshan 528400, China; 3 University of Chinese Academy of Sciences, Beijing 100049, China; 4 State Key Laboratory of Quality Research in Chinese Medicine, Institute of Chinese Medical Sciences, University of Macau, Taipa, Macao 999078, China; 5 School of Chinese Materia Medica, Nanjing University of Chinese Medicine, Nanjing 210023, China; 6 School of Pharmaceutical Sciences, Guizhou Medical University, Guizhou 561113, China; 7 School of Pharmaceutical Sciences, Southern Medical University, Guangzhou 510515, China

**Keywords:** histidine kinase, BaeS, two component system, antibiotic stress response, multidrug efflux

## Abstract

*Escherichia coli*, a facultative anaerobic Gram-negative member of the Enterobacteriaceae, is an increasingly important opportunistic pathogen driven in part by rising resistance to clinically important antibiotics. Regulation of multidrug efflux systems by two-component signal transduction pathways, particularly the BaeSR system, plays a central role in this process. However, the functional residues governing signal transduction through the sensor kinase BaeS remain incompletely defined. In this study, we integrated domain prediction, homology-guided site-directed mutagenesis, in vitro protein purification, autophosphorylation assays, and reverse-transcription quantitative polymerase chain reaction (RT-qPCR)-based transcriptional analysis of selected BaeSR-regulated genes to delineate key residues required for BaeS function. Sequence analysis identified His250 as a candidate autophosphorylation site and Asn364 as a conserved residue within the catalytic domain. Biochemical characterization of purified wild-type BaeS and an H250A mutant demonstrated that His250 is indispensable for autophosphorylation. Consistently, RT-qPCR analysis showed that BaeS activation markedly induced the transcription of BaeSR-regulated efflux-associated genes, whereas genetic deletion of *baeS* or selective disruption of kinase activity by the N364A mutation abolished this response. Together, these findings establish His250 as a key residue for BaeS autophosphorylation and identify Asn364 as essential for inducible BaeSR signaling and activation of resistance-associated target genes, thereby establishing an experimental framework for elucidating BaeSR-mediated efflux regulation and informing future studies of resistance regulatory networks and potential intervention strategies centered on key signaling nodes.

## 1. Introduction

Bacterial adaptation to antibiotic stress depends on sophisticated regulatory networks that enable rapid sensing of environmental challenges and coordinated transcriptional responses. Among these networks, two-component systems (TCSs) represent a principal signaling mechanism by which bacteria detect extracellular cues and modulate gene expression programs critical for survival under antimicrobial pressure [1,2]. TCSs regulate diverse physiological processes, including antibiotic resistance, virulence, quorum sensing, biofilm formation, metal homeostasis, motility, and stress adaptation [3,4], rendering them attractive targets for antimicrobial intervention [5,6]. In members of the Enterobacteriaceae, multiple TCSs have been implicated in antibiotic resistance, including BaeSR, CpxAR, EnvZ/OmpR, EvgAS, PhoPQ, PmrAB, and RcsBCD [7]. A central determinant of antimicrobial susceptibility in Gram-negative bacteria is the expression of outer membrane porins and multidrug efflux pumps, both of which are frequently regulated by TCS-mediated signaling pathways [1,8]. Among these systems, the BaeSR TCS plays a particularly prominent role in both intrinsic and inducible resistance responses in enteric bacteria [9].

BaeSR consists of the membrane-associated sensor histidine kinase BaeS and the cytoplasmic response regulator BaeR. In response to envelope-associated stress signals, BaeS undergoes autophosphorylation and subsequently transfers the phosphoryl group to a conserved aspartate residue in BaeR. The phosphorylated response regulator (BaeR~P) functions as a dimeric transcriptional activator, inducing the expression of resistance-associated genes, including the multidrug efflux operons *mdtABCD* and *acrD*, as well as the periplasmic stress protein gene *spy* [9,10,11,12]. Through this regulatory cascade, BaeSR enhances bacterial tolerance to diverse toxic compounds and antibiotics, underscoring its central role in antibiotic resistance regulation in *Escherichia coli* K12 [13]. The BaeSR system has been implicated in multiple antibiotic-associated phenotypes in Gram-negative bacteria. For example, in *E. coli*, BaeSR contributes to novobiocin-associated resistance phenotypes [14]. In *Salmonella enterica* serovar Typhimurium, BaeSR has been shown to participate in ciprofloxacin-induced *mdtA* expression and is also associated with ceftriaxone-related phenotypes [15]. More recently [13], temocillin exposure has been reported to select for *baeS* mutations in *E. coli*, suggesting that altered BaeSR signaling may contribute to resistance-associated phenotypes through modulation of downstream efflux-related genes such as *mdtABC* and *acrD*. However, because antibiotic exposure may simultaneously impose growth inhibition and trigger multiple overlapping stress responses, the best-characterized activators of the BaeSR pathway among experimentally validated inducers are non-antibiotic pleiotropic compounds, particularly sodium tungstate, which is considered a more suitable and experimentally tractable stimulus [16,17]. Compared with other candidate compounds, sodium tungstate is one of the best-characterized and most reproducible stimuli for pathway-specific analysis of BaeSR signaling. Therefore, sodium tungstate was used in the present study as a defined inducer of BaeSR signaling.

BaeS belongs to the HisKA family of histidine kinases, which includes well-characterized members such as *E. coli* EnvZ [10]. Proteins within this family typically possess dual kinase and phosphatase activities, enabling tight control of response regulator phosphorylation states. These activities can be functionally dissected through site-directed mutagenesis of conserved residues that govern catalysis and phosphotransfer [10]. Although genetic and transcriptional studies have firmly established the role of BaeSR in efflux pump regulation and antibiotic tolerance, the molecular determinants underlying BaeS kinase activity remain incompletely defined [7]. In particular, the specific residues responsible for BaeS autophosphorylation and catalytic function have not been systematically characterized, limiting mechanistic insight into how resistance-associated transcriptional programs are controlled at the level of the sensor kinase. Given the increasing interest in TCSs as therapeutic targets, elucidating the functional architecture of BaeS is of both mechanistic and translational significance.

In this study, we combined I-TASSER-based structural prediction with homology guided sequence alignment to identify His250 as a putative autophosphorylation site and Asn364 as a conserved residue potentially involved in catalytic regulation [18,19]. Full-length BaeS and the H250A mutant were expressed and purified, and their phosphorylation states were examined using in vitro autophosphorylation assays coupled with Phos-tag analysis [20,21]. In parallel, *E. coli* K12 was used as the parental strain to construct a *baeS* deletion mutant (Δ*baeS*), a low-copy, native-promoter complementation strain (WTc), and a kinase-site mutant carrying the N364A substitution. Transcriptional responses of BaeSR regulated resistance genes including *spy* [22,23], *mdtA* [24], and *acrD* [25] were subsequently analyzed by reverse transcription quantitative polymerase chain reaction (RT-qPCR) under inducing conditions to evaluate the functional consequences of kinase site mutations [16]. Together, these analyses delineate key residues contributing to BaeS autophosphorylation and kinase activity and clarify their roles in BaeSR mediated regulation of antibiotic resistance.

## 2. Materials and Methods

### 2.1. Bioinformatics Analysis

Conserved domains of the BaeS sensor kinase and the response regulator BaeR were identified using the SMART conserved domain database (http://smart.embl.de/). The predicted structural architecture of BaeS was generated using the I-TASSER protein structure prediction server [18,19]. Based on this prediction, the most similar structural homolog VicK [26], and the most thoroughly studied member of the HisKA family, EnvZ [8,27], were identified. The cytoplasmic-domain sequences of BaeS, VicK, and EnvZ were subjected to multiple sequence alignment using MEGA version 11.0.13 [28] and visualized with Jalview version 2.11.5.1, enabling the identification of conserved residues and candidate functional motifs, including putative autophosphorylation and catalytic residues for subsequent site-directed mutagenesis.

To further evaluate the conservation of these candidate residues in closely related bacteria, BaeS orthologs from representative Enterobacteriaceae, including *Salmonella enterica*, *Klebsiella pneumoniae*, *Enterobacter cloacae*, *Citrobacter freundii*, *Cronobacter sakazakii*, and *Raoultella ornithinolytica*, were additionally collected and aligned. The cytoplasmic-domain sequences were analyzed by multiple sequence alignment using MEGA version 11.0.13 and visualized with Jalview version 2.11.5.1.

### 2.2. Recombinant Plasmids, Bacterial Strains and Growth Conditions

The strains and plasmids used in this study are listed in Table 1. The designed BaeS and BaeS-H250A coding sequences were synthesized and cloned by GenScript (Nanjing, China) into the pGEX-6P-1 vector using BamHI and NotI restriction sites for protein expression.

In addition, we designed an expression cassette containing the *baeS* gene and its native promoter region from *Escherichia coli* MG1655, with the promoter region defined as the 500 bp sequence upstream of the start codon. These DNA fragments were synthesized by GenScript and integrated into the MCS1 region of a modified low-copy-number pACYCDuet-1 backbone. Briefly, the original T7-driven expression module in the MCS1 region was replaced with a synthetic insert comprising the native *baeS* promoter, the full-length *baeS* coding sequence, a C-terminal 6× His tag, and a stop codon, while the downstream T7 terminator was retained. Because only a single expression cassette was required for the complementation assays, the T7 promoter module in the MCS2 region was also removed. The resulting plasmid, BaeS-pACYCDuet-1, was constructed by GenScript using CloneEZ seamless cloning, and the mutant plasmid BaeS-N364A-pACYCDuet-1 was generated in the same manner.

The *E. coli* MG1655 strain was purchased from Shanghai Weidi Biotechnology Co., Ltd. (Shanghai, China) and used as the parental wild-type strain (WTp). The *baeS* deletion mutant (Δ*baeS*) was generated in *E. coli* MG1655 using the GenScript microbial CRISPR genome-editing system. Guide RNAs targeting the *baeS* locus and a donor construct carrying 5′ and 3′ homologous arms were introduced into the host strain for genome editing based on the deletion design shown in Figure S1. Candidate edited colonies were screened by colony PCR and further validated by Sanger sequencing (Figures S2 and S3). Using primers BaeS-F1 and BaeS-R2, the expected amplicon sizes were 2360 bp for the wild-type allele and 1021 bp for the knockout allele. Sequence analysis of the positive clone confirmed a 1339 bp internal deletion within the *baeS* coding region (Figure S3). For genetic complementation, a low-copy plasmid carrying *baeS* under the control of its native promoter (BaeS-pACYCDuet-1) was introduced into the Δ*baeS* strain by GenScript. The complemented strain was referred to as the complemented wild-type strain (WTc) in subsequent experiments. Similarly, the kinase deficient mutant strain (N364A) was constructed by introducing BaeS-N364A-pACYCDuet-1 into the Δ*baeS* background by GenScript. *E. coli* cells were cultured in MOPS minimal medium supplemented with thiamine (1 μg/mL) and uracil (20 μg/mL) at 25 °C [29].

### 2.3. Expression and Purification of Full-Length BaeS and BaeS-H250A Proteins

BaeS and BaeS-H250A were expressed in *Escherichia coli* C43(DE3) pLysS carrying BaeS-pGEX-6P-1 and BaeS-H250A-pGEX-6P-1, respectively. Overnight cultures were inoculated into 1 L fresh LB medium and grown to an OD_600_ of approximately 0.8. Protein expression was then induced with 0.15 mM IPTG at 16 °C and 150 rpm for 18 h. The induced cultures (O_D600_ ≈ 2.0) were harvested by centrifugation at 5100× *g* for 1 h at 4 °C. Cell pellets from each liter of culture were resuspended in 50 mL ice-cold lysis buffer (1× PBS, 1% Triton X-100 [Beyotime Biotechnology, Shanghai, China], 1.5 mg/mL lysozyme, 1 mM DTT, and 1 mM PMSF, pH 8.0) and disrupted by sonication on ice (600 W, 3 s on/1 s off, total 15 min). The lysates were then centrifuged at 10,000× *g* for 45 min at 4 °C, and the supernatants were collected and incubated with 2 mL Glutathione Sepharose resin (Yeasen, Shanghai, China) for 2 h at 4 °C. The resin slurry was then transferred to a gravity-flow column and washed with buffer A (1× PBS, 1 mM DTT, 0.25% Triton X-100 [Beyotime Biotechnology, Shanghai, China], pH 8.0) to remove nonspecifically bound proteins. Bound proteins were sequentially eluted with buffer B (50 mM Tris-HCl, 150 mM NaCl, 1 mM DTT, 0.25% Triton X-100 [Beyotime Biotechnology, Shanghai, China], pH 8.0) containing 10 mM and then 20 mM reduced glutathione. Fractions containing the target protein were further purified by size-exclusion chromatography using buffer A as the running buffer at a flow rate of 0.4 mL/min. Under these conditions, full-length BaeS was reproducibly recovered in the detergent-solubilized fraction.

### 2.4. BaeS and BaeS-H250A Self-Phosphorylation Reaction In Vitro

To study the autophosphorylation activity of BaeS and its mutant BaeS-H250A, the purified proteins were quantified by the BCA assay and then incubated with 5 μM ATP in kinase reaction buffer (25 mM Tris-HCl, 50 mM KCl, 1 mM CaCl_2_, 1 mM MgCl_2_, pH 8.0) at 37 °C for 60 min [30]. After the reaction, 3× loading buffer was added to terminate the reaction, followed by the addition of 10 mM ZnCl_2_ solution to stabilize the phosphorylation state. The samples were then used for subsequent experimental analysis.

### 2.5. Analysis of BaeS Autophosphorylation by Zn^2+^-Phos-tag™ SDS-PAGE

To detect the phosphorylation state of the BaeS and BaeS-H250A, this study employed a Zn^2+^-Phos-tag™-based Western blot technique. After autophosphorylation reactions, BaeS and BaeS-H250A were loaded onto a 6% Phos-tag™ SDS-polyacrylamide gel containing 50 μM Phos-tag™ acrylamide (Wako, Osaka, Japan) and 100 μM ZnCl_2_ for electrophoretic separation [30]. Following electrophoresis, proteins were transferred to 0.22 μm PVDF membranes (Merck Millipore, Darmstadt, Germany) and probed with an anti-His tag primary antibody (Abcam, Cambridge, UK; 1:1000 dilution) to detect phosphorylated and unphosphorylated forms of BaeS. A HRP-conjugated goat anti-mouse IgG (H + L) (Beyotime Biotechnology, Shanghai, China; 1:1000 dilution) was used as the secondary antibody. Membranes were blocked with 5% bovine serum albumin (BSA) (Macklin, Shanghai, China) in Tris-buffered saline containing 0.1% Tween-20 (TBST) for 2 h at room temperature to minimize nonspecific binding and improve detection specificity for membrane-associated and phosphorylated proteins. Protein bands were visualized by chemiluminescence imaging, and phosphorylation was assessed based on the characteristic mobility retardation of phosphorylated BaeS on Phos-tag™ gels.

### 2.6. Bacterial Culture and Induction Conditions

WTp, WTc, Δ*baeS*, and N364A *Escherichia coli* strains were grown in antibiotic-free MOPS minimal medium at 37 °C to the mid-logarithmic phase. Cultures were then divided equally into basal and induction groups. For induction, cultures were treated with 5 mM Na_2_WO_4_ [16] and 5 μM ATP and incubated for an additional 45 min, whereas the basal group received no treatment and was incubated for the same duration.

### 2.7. RNA Extraction and RT-qPCR Analysis

Following treatment, 0.5 mL of each culture was harvested and immediately mixed with 1 mL of RNeasy Bacterial Protect Reagent (Qiagen, Hilden, Germany). Samples were vortexed for 5 s, incubated at room temperature for 5 min, and centrifuged at 5500× *g* for 10 min. Cell pellets were rapidly frozen in a dry ice–ethanol bath and stored at −80 °C until RNA extraction.

Total RNA was isolated using the RNeasy Mini Kit (Qiagen, Hilden, Germany) according to the manufacturer’s instructions, including on-column DNase I digestion to remove residual genomic DNA. Purified RNA samples were further treated with DNase I using the DNA-free™ DNase Treatment and Removal Kit (Ambion, Austin, TX, USA) for 30 min to ensure complete elimination of DNA contamination. RNA concentration and purity were determined using a NanoDrop spectrophotometer (Thermo Scientific, Waltham, MA, USA), assuming an extinction coefficient of 27 (ng mL^−1^)^−1^ cm^−1^.

First-strand cDNA synthesis was performed using the SuperScript™ III Reverse Transcriptase kit (Invitrogen, Carlsbad, CA, USA) with random hexamer primers and total RNA as the template. Approximately 50 ng of synthesized cDNA was used as template for each 20 μL qPCR reaction. Transcript levels of *spy*, *mdtA*, and *acrD* were quantified by RT-qPCR using gene-specific primers listed in Table 2. The housekeeping gene *gapA* was used as an internal reference for normalization.

RT-qPCR was performed in triplicate using SYBR Green qPCR SuperMix (Invitrogen, Carlsbad, CA, USA) on a Mastercycler^®^ ep realplex real-time PCR system (Eppendorf, Hamburg, Germany). Relative gene expression levels were calculated using standard comparative quantification methods. Data are presented as mean ± standard deviation (SD). Statistical significance was evaluated using ordinary one-way ANOVA followed by Tukey’s multiple comparisons test. A value of *p* < 0.05 was considered statistically significant.

### 2.8. Membrane Protein Extraction and Western Blot Analysis

Cultures of the empty vector strain (EV), WTc, and N364A grown under the same conditions as described in Section 2.6 were used for Western blot analysis. After treatment, cells were harvested by centrifugation and resuspended in membrane extraction buffer (50 mM Tris-HCl, 150 mM NaCl, 1 mM PMSF, 1.5 mg/mL lysozyme, and a protease inhibitor cocktail at 1× final concentration, pH 8.0). The suspensions were incubated on ice for 30 min and subsequently disrupted by sonication on ice (300 W, 3 s on/5 s off, total sonication time of 5 min) until the lysates were clarified. Unbroken cells and large debris were removed by centrifugation at 4000× *g* for 10 min at 4 °C, and the supernatants were collected. Total membrane fractions were then obtained by centrifugation of the supernatants at 10,000× *g* for 1 h at 4 °C. The resulting membrane pellets were resuspended in 100 μL of solubilization buffer (50 mM Tris-HCl and 2% Triton X-100 [Beyotime Biotechnology, Shanghai, China], pH 8.0). Protein concentrations were determined using a BCA protein assay.

Equal amounts of protein were mixed with SDS loading buffer, incubated at 70 °C for 10 min, separated by SDS-PAGE, and transferred onto 0.22 μm PVDF membranes (Merck Millipore, Darmstadt, Germany). After transfer, the membrane was cut according to the molecular weights of the target proteins and probed separately. Membranes were blocked with 5% BSA (Macklin, Shanghai, China) in TBST for 2 h at room temperature. BaeS was detected using a mouse anti-His tag primary antibody (Abcam, Cambridge, UK; 1:1000 dilution) and an HRP-conjugated goat anti-mouse IgG (H + L) secondary antibody (Beyotime Biotechnology, Shanghai, China; 1:1000 dilution). SecG served as the loading control and was detected using a rabbit anti-SecG primary antibody (MyBioSource, San Diego, CA, USA; 1:1000 dilution), followed by an HRP-conjugated goat anti-rabbit IgG (H + L) secondary antibody (Beyotime Biotechnology, Shanghai, China; 1:1000 dilution) [32]. Protein signals were visualized using Meilunbio^®^ West Femto Maximum Sensitivity Substrate (Meilunbio, Dalian, China) mixed at a 1:1 ratio. Band intensities were quantified by densitometric analysis using ImageJ software version 1.54p. The BaeS signal in each lane was normalized to the corresponding SecG signal, and the resulting BaeS/SecG ratio was used for statistical analysis. Data are presented as mean ± SD from three independent biological replicates. Differences between WTc and N364A were evaluated using an unpaired two-tailed Student’s *t*-test.

## 3. Results

### 3.1. Conserved Domain Analysis and Design of Functional BaeS Mutants

**Figure 1:**
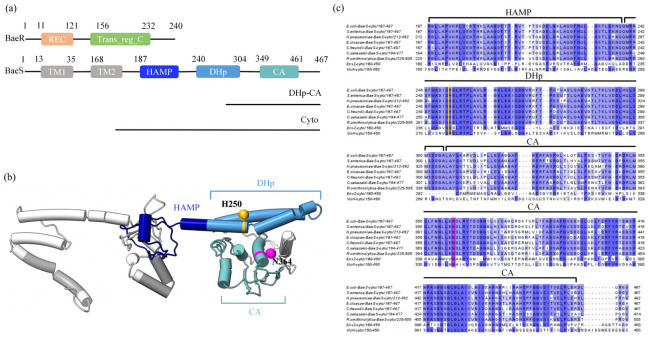
Domain organization and structural analysis identify candidate functional residues in BaeS. (**a**) Schematic domain architecture of BaeS and BaeR. BaeS contains two transmembrane regions (TM1 and TM2), a HAMP linker domain, a dimerization and histidine phosphotransfer (DHp) domain, and a catalytic ATP-binding (CA) domain. BaeR contains an N-terminal receiver (REC) domain and a C-terminal transcriptional regulatory domain (Trans_reg_C). (**b**) Sequence alignment of the intracellular catalytic regions of BaeS with those of the homologous histidine kinases EnvZ and VicK. The HAMP linker, DHp, and CA domains are indicated. Residues subjected to mutagenesis in this study and implicated in autophosphorylation or kinase activity are boxed in yellow and magenta, respectively. (**c**) Structural modeling of BaeS protein. The HAMP, DHp, and CA domains are labeled and shown in midnightblue, steelblue and cadetblue, (TM1 and TM2 are shown in gray; the other monomer is shown in dark gray), respectively. Amino acid residues that were mutated in this study and that are predicted to be involved in BaeS autophosphorylation and kinase activities are mapped on the structural model as goldenrod and magenta, respectively, and labeled.

Conserved-domain prediction and homology modeling revealed the structural organization of BaeS, as shown in Figure 1a. BaeS contains two transmembrane helices (residues 13–35 and 168–190), followed by a typical HAMP domain (residues 187–239), a HisKA (DHp) domain (residues 240–304) responsible for autophosphorylation, and a C-terminal catalytic ATP-binding (CA) domain (residues 349–461). These features indicate that BaeS belongs to the HisKA family of histidine kinases. In parallel BaeR consists of an N-terminal receiver (REC) domain and a C-terminal transcriptional regulatory domain (Trans_reg_C).

To identify functionally important residues for mutagenesis, the cytoplasmic domain sequence alignment was performed between BaeS and representative HisKA family members, including EnvZ, serving as one of the best characterized histidine kinases, and VicK, the closest structural homolog of BaeS based on I-TASSER predictions. Multiple sequence alignment is shown in Figure 1b. To further assess the conservation of these residues in closely related bacteria, we additionally performed a multiple sequence alignment of the cytoplasmic domains of BaeS orthologs from six representative Enterobacteriaceae. The analysis showed that these residues are conserved or highly conserved among the aligned BaeS homologs, further supporting their potential functional importance.

Based on the conserved motifs revealed by this analysis, two conserved residues were selected for site-directed mutagenesis. His250 is located in the DHp domain and aligns with the canonical autophosphorylatable histidine in homologous HisKA-family kinases, such as EnvZ. This suggests that His250 may serve as the core phospho-accepting residue of BaeS [32]. Asn364, located in the CA domain, was selected because it corresponds to the conserved Asn347 residue of EnvZ, a catalytic-site residue previously reported to be critical for ATP-dependent kinase activity, and this position is also highly conserved in VicK [33,34]. Thus, His250 was chosen as the predicted autophosphorylation site, whereas Asn364 was selected as a candidate residue required for kinase activity. Both residues were individually substituted with alanine. The predicted structural model of BaeS and the spatial locations of the mutated residues are shown in Figure 1c.

### 3.2. Expression and Purification of Full-Length BaeS and the BaeS-H250A Mutant

**Figure 2:**
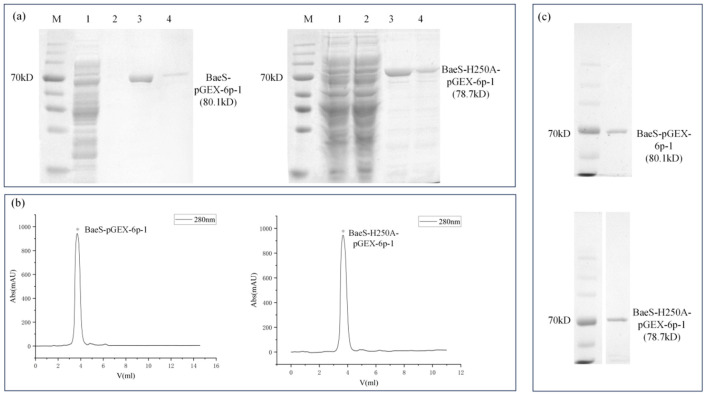
Purification of full-length BaeS and the BaeS-H250A mutant. (**a**) SDS-PAGE analysis of BaeS and BaeS-H250A elution by reduced glutathione buffer. M: Prestained protein ladder, 1: Flow through of Glutathione Sepharose chromatography column, 2: The column was washed with buffer A removing nonspecifically bound proteins, 3: Fraction of washing with buffer B containing 10 mM reduced glutathione, 4: Fraction of washing with buffer B containing 20 mM reduced glutathione. (**b**) Size exclusion chromatogram of BaeS and BaeS-H250A. The asterisks indicate the elution peaks collected for subsequent analysis. (**c**) SDS-PAGE results of BaeS and BaeS-H250A eluted peaks. M: Prestained protein ladder, Purified BaeS and BaeS-H250A, around 80.1 kD and 78.7 kD, respectively.

BaeS and BaeS-H250A were purified by GST affinity chromatography using reduced glutathione for elution. SDS-PAGE analysis showed that both proteins were efficiently eluted at a concentration of 10 mM reduced glutathione (Figure 2a). The eluted fractions containing BaeS or BaeS-H250A were separately collected, concentrated, and further purified by size-exclusion chromatography. As shown in Figure 2b, both proteins eluted as distinct major peaks, corresponding to their respective molecular weights. The peak fractions of BaeS and BaeS-H250A were collected and analysed (Figure 2c).

### 3.3. His250 Is Required for BaeS Autophosphorylation In Vitro

**Figure 3:**
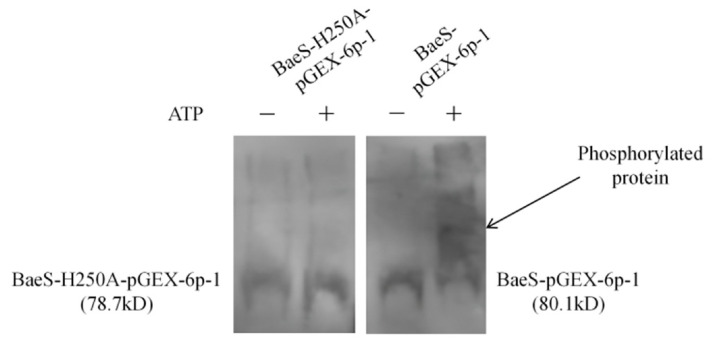
Characterization of the phosphorylation levels of BaeS and BaeS-H250A proteins in vitro. The positions of molecular mass standards (kDa) are indicated on the right. In the absence of ATP, both WT BaeS and the H250A mutant migrated to a similar position corresponding to the unphosphorylated state. Upon addition of ATP, WT BaeS exhibited a pronounced upward band shift.

Through preliminary experiments comparing the separation efficiencies of 20, 30, 40, and 50 μM Phos-tag™ Acrylamide, we ultimately selected 50 μM Phos-tag™ acrylamide for the protein autophosphorylation characterization experiment. The results of the Western blot analysis are shown in Figure 3. In the presence of 5 μM ATP, purified BaeS and BaeS-H250A were subjected to in vitro autophosphorylation assays. Purified BaeS underwent autophosphorylation, while no autophosphorylation was observed for BaeS-H250A under the same conditions. These results suggest that BaeS autophosphorylation requires the His 250 residue, indicating that His 250 is the autophosphorylation site in the histidine kinase BaeS.

### 3.4. Asn364 Is Essential for BaeS-Dependent Efflux Gene Induction

**Figure 4:**
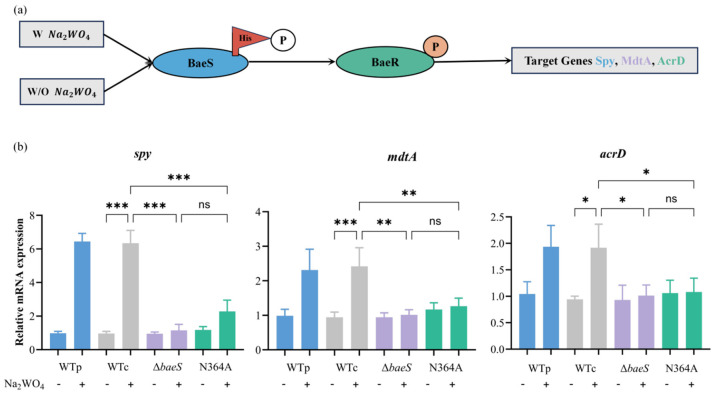
Asn364 is essential for BaeS-dependent induction of resistance-associated target genes. (**a**) Schematic representation of the BaeSR TCS signaling pathway. (**b**) Na_2_WO_4_-induced activation of BaeSR-dependent gene expression requires BaeS kinase activity. Transcript levels were normalized to *gapA* and are presented relative to the untreated WTp strain. Data are shown as mean ± SD (*n* = 3). Statistical significance was evaluated using ordinary one-way ANOVA followed by Tukey’s multiple comparisons test. * *p* < 0.05, ** *p* < 0.01, *** *p* < 0.001; ns, not significant.

RT-qPCR analysis showed that sodium tungstate (Na_2_WO_4_) treatment significantly increased the transcript levels of *spy*, *mdtA*, and *acrD* in the parental wild-type strain (WTp) compared with the corresponding basal condition, indicating activation of the BaeSR two-component system (Figure 4b). In contrast, Na_2_WO_4_ induction failed to elicit significant transcriptional changes in these genes in the *baeS* deletion mutant (Δ*baeS*), serving as a negative control. Notably, the BaeS kinase-site mutant (N364A) exhibited a transcriptional profile similar to that of Δ*baeS*, with no significant induction of *spy*, *mdtA* and *acrD* upon Na_2_WO_4_ treatment and markedly reduced expression relative to induced WTp. In contrast, the wild-type complementation strain (WTc) restored Na_2_WO_4_ responsive induction of all three genes to levels comparable to those of WTp.

**Figure 5:**
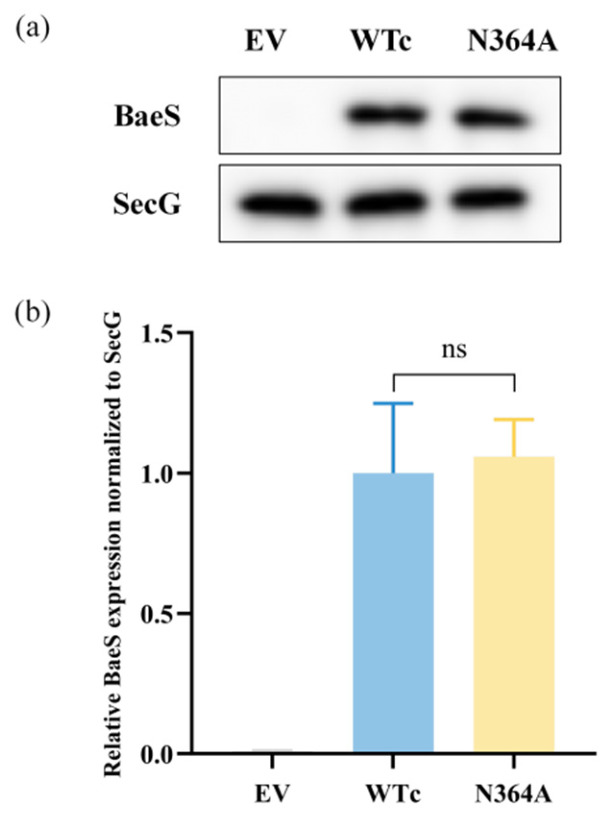
Expression of BaeS WT and BaeS N364A mutant. (**a**) Representative Western blot showing BaeS protein levels in the empty-vector control (EV), the wild-type complementation strain (WTc), and the kinase-site mutant (N364A). SecG was used as the loading control. (**b**) Densitometric quantification of BaeS expression normalized to SecG. Data are presented as mean ± SD from three independent biological replicates. Statistical significance between WTc and N364A was evaluated using an unpaired two-tailed Student’s *t*-test. ns, not significant.

To exclude the possibility that the defective transcriptional response of the N364A mutant resulted from impaired BaeS expression rather than loss of function, we further compared BaeS protein levels in the complemented strains by Western blot analysis. As shown in Figure 5a, a clear BaeS signal was detected in both WTc and N364A, whereas no specific signal was observed in the empty-vector control (EV). After normalization to the inner membrane protein SecG, no significant difference in BaeS abundance was observed between WTc and N364A (Figure 5b). These results indicate that the impaired induction of downstream BaeSR-regulated genes in the N364A mutant is not attributable to reduced protein expression, but rather reflects a functional defect in BaeS-mediated signaling.

Taken together, these data demonstrate that Asn364 is indispensable for BaeS-mediated signaling and for the induction of downstream efflux-related genes under tungstate stimulation.

## 4. Discussions

Unlike well-characterized sensor histidine kinases such as EnvZ [28], although the BaeSR TCS plays a pivotal role in multidrug resistance regulation, the functional residues governing BaeS autophosphorylation and downstream signaling have remained largely unexplored [35]. To address this gap, we employed an integrated strategy combining conserved domain prediction, homology-guided site-directed mutagenesis, in vitro biochemical characterization, and transcriptional analysis under physiological bacterial conditions. This approach enabled us to directly link specific residues within BaeS to defined biochemical activities and in vivo signaling outputs, thereby bridging the gap between sequence conservation, kinase activity, and resistance-associated gene regulation. Notably, we established a robust system for the soluble expression and high-purity purification of full-length BaeS from *Escherichia coli*, overcoming a major technical challenge commonly associated with membrane-embedded histidine kinases. Using in vitro autophosphorylation assays in combination with mutagenesis, we unambiguously identified His250 as the critical autophosphorylation site of BaeS. Consistent with the conserved role of the H-box histidine in HisKA-family kinases, wild-type BaeS exhibited strong ATP-dependent autophosphorylation activity, whereas substitution of His250 with alanine completely abolished the phosphorylation signal, confirming its essential role in BaeS catalytic function. Optimization of expression host selection and detergent conditions further enabled the recovery of enzymatically active BaeS, providing a solid experimental foundation for future structural and mechanistic studies, including phosphotransfer analyses with the response regulator BaeR.

Beyond the autophosphorylation site, we further demonstrate that conserved residues Asn364 within the catalytic ATP-binding (CA) domain are indispensable for BaeS-dependent signaling in vivo. Substitution of these residues markedly attenuated Na_2_WO_4_ induced transcription of the efflux-associated genes *spy*, *mdtA*, and *acrD*, closely phenocopying the Δ*baeS* mutant. Importantly, restoration of inducible signaling in a low-copy, native-promoter complementation strain excluded altered expression levels as the cause of the observed defects, indicating that these phenotypes reflect genuine impairments in BaeS kinase function. Notably, although transcriptional induction was strongly reduced in the N364A mutant, residual responsiveness relative to the deletion strain suggests that additional regulatory elements may contribute to fine-tuning BaeS activity. While ATP-binding kinetics were not directly measured in this study, the localization of Asn364 within the conserved CA domain supports a model in which these residues help maintain a catalytically competent conformation required for efficient signal output. The concordance between in vitro autokinase defects and in vivo transcriptional responses underscores the physiological relevance of BaeS catalytic integrity.

In summary, our findings elucidate key molecular determinants underlying BaeS-mediated signal transduction and position BaeS as a promising sensor-kinase-level target for strategies aimed at disrupting multidrug resistance in Enterobacteriaceae.

## Figures and Tables

**Table 1:** Strains and plasmids used in the study.

**Strains or Plasmids**	**Description**
**Strains**	
*E. coli* MG1655 WTp	Parental wild-type strain of *E. coli* MG1655
Δ*baeS*	*baeS*-deletion mutant constructed in the *E. coli* MG1655 wild-type background
WTc	*E. coli* MG1655 WTp trans-complemented strain carrying BaeS-pACYCDuet-1
N364A	*E. coli* MG1655 WTp trans-complemented strain carrying BaeS-N364A-pACYCDuet-1
**Plasmids**	
BaeS-pGEX-6P-1	GST fusion protein expression vector
BaeS-H250A-pGEX-6P-1	GST fusion protein expression vector
BaeS-pACYCDuet-1	Low-copy-number complementation vector carrying *baeS* under its native promoter
BaeS-N364A-pACYCDuet-1	Low-copy-number complementation vector carrying *baeS*-N364A under its native promoter

**Table 2:** Target genes and primers used in the study.

Target Gene	Primer	Sequence (5′ to 3′)	Expected Amplicon Size (bp)	Reference
*spy*	Spy-F	GCAACAGTACCGCAGTGAAA	~120	[31]
	Spy-R	CGTCAACATAGCCGATACCA		
*mdtA*	MdtA-F	TTAATAATCAGGATGATGCGCTGT	~150	[31]
	MdtA-R	CACCACTTTCTGACTGTCCTGAAT		
*acrD*	AcrD-F	TTTTTTGTGCCCGACACCTCG	~140	[11]
	AcrD-R	AAATCTATAACGATATGTAGA		
*gapA*	GapA-F	ACTTACGAGCAGATCCTGCT	~120	This study
	GapA-R	TACCATGAGATCACGACGCG		

## Data Availability

The data presented in this study are available in the article and Supplementary Materials.

## Supplementary Materials

The following supporting information can be downloaded at: https://www.mdpi.com/article/10.3390/microorganisms14051031/s1, Figure S1: Verification of the *baeS* deletion design in the *Escherichia coli* MG1655 host strain; Figure S2: Colony PCR screening of independent edited colonies for identification of the Δ*baeS* mutant; Figure S3: Sanger sequencing verification of the deletion junction in the Δ*baeS* mutant.
