# Supplementary material for: Functional Characterization of the Histidine Kinase BaeS Reveals Critical Residues for BaeSR-Dependent Stress Signaling in Escherichia coli
Source: Microorganisms. 2026 May 1;14(5):1031. doi: 10.3390/microorganisms14051031 (PMC13209484; doi:10.3390/microorganisms14051031)

Supplementary Materials

Paper title: Functional Characterization of the Histidine Kinase BaeS Reveals Critical Residues for BaeSR-Dependent Stress Signaling in *Escherichia coli.*

This supplementary document contains host strain information, colony PCR screening data, and deletion-junction sequencing verification for construction of the seamless Δ*baeS* mutant.

Figure S1 Verification of the *baeS* deletion design in the *Escherichia coli* MG1655 host strain.

The parental host strain used for genome editing was *Escherichia coli* MG1655. The sequence shown corresponds to the wild-type *baeS* locus in this strain, and the underlined region indicates the segment deleted during construction of the Δ*baeS* mutant.

>gene sequence of *baeS*

tttgccagagtgccgaacgatacgcatcaaaatgtagctatttcgcggcgaaaaggagcgcgcaatgaagttctggcgacccggtat
taccggcaaactgtttctggcgattttcgccacctgcattgtcttgctgatcagtatgcactgggcggtgcgtatcagttttgagcgtggctttattg
attacatcaagcatggtaatgaacagcgattacaactgttaagtgatgcgcttggcgagcagtatgcgcagcatggcaactggcgcttcctgcg
caacaatgatcgctttgtctttcagatcctgcgttcatttgaacacgataattcggaagataaacccggcccgggtatgccaccgcacggctgg
cgtacccagttctgggtggttgatcaaaacaacaaagtgctggttggtccgcgagcgccgattccacctgacggtacacggcgacccattctg
gtcaacggtgcggaagttggcgcggtgatcgcctcccccgttgagcggttaacgcgcaatactgatatcaatttcgataaacaacagcggca
aaccagctggttgattgtcgccctggcaacgttactcgcggcacttgccacttttctgctggcgcgcggtttactggcaccggtaaaacgacttg
tcgatggcacgcacaaactggcggcgggcgatttcactacccgcgtaacgcccaccagtgaagatgaactgggcaaactggcgcaagactt
caaccagcttgccagcacactggagaaaaaccagcaaatgcggcgcgattttatggccgatatttctcacgaactgcgtacgccattagcggt
gctgcgcggtgaactggaagccattcaggatggcgtgcgtaaattcacgccggagacggtggcgtctttacaggcggaggtcggtacactg
accaaactggttgacgatctccatcagttgtcgatgtctgatgaaggcgctctcgcctatcaaaaagcaccggtagatttgatcccactgctgga
agtggcgggcggcgcatttcgcgaacgattcgccagtcgtggcctgaaactgcaattttccctgccagacagtattaccgtatttggcgatcgc
gaccgtttaatgcagttattcaataacttactggaaaacagcctgcgctacactgacagcggcggcagcctgcaaatctctgccgggcagcgc
gacaaaacggtgcgcctgacctttgccgacagtgcgccaggtgtcagtgacgatcagctacaaaaattgtttgaacgtttttatcgcaccgaag
gttcccgcaaccgtgccagcggcggttccgggctggggctggcgatttgcctgaacattgttgaagcacataatggtcgcattattgctgccca
ttcgccttttggcggggtaagcattacagtagagttaccgctggaacgggatttacagagagaagt**atgaccgagttaccaatcgacgaaa
acacaccgcgtattttgatc**

Figure S2 Colony PCR screening of independent edited colonies for identification of the Δ*baeS* mutant.

Colony PCR screening was performed on eight independent edited colonies using primers BaeS-F1 and BaeS-R2, with wild-type *E. coli* MG1655 used as the control. Lane M: MF024 1 kb DNA ladder; lanes 1-8: independent edited colonies (KO1-KO8); lane 9: wild-type MG1655. The expected PCR product sizes were 2360 bp for the wild-type allele and 1021 bp for the Δ*baeS* allele. Colony KO2, which yielded the expected knockout-sized amplicon, was selected for further sequencing confirmation.

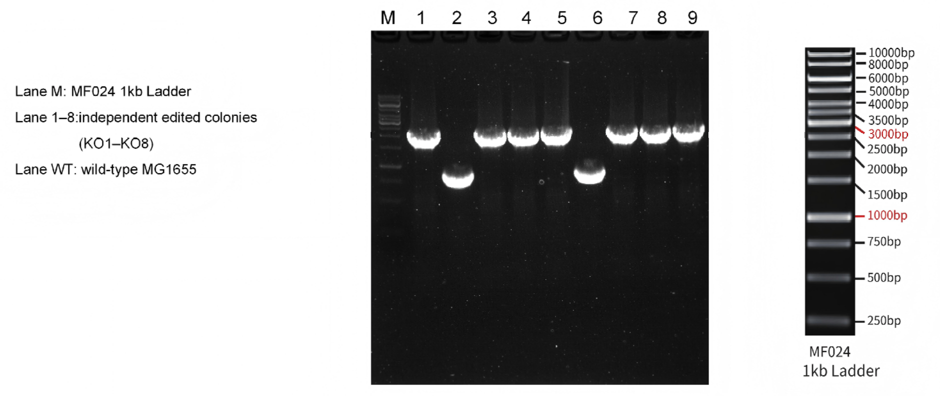

Figure S3 Sanger sequencing verification of the deletion junction in the Δ*baeS* mutant.

The PCR fragment amplified from the positive edited colony (KO2) was gel-purified and subjected to Sanger sequencing using primer BaeS-F1. Sequence alignment against the wild-type *baeS* locus using SnapGene confirmed a 1339-bp internal deletion within the *baeS* coding region. The schematic diagram indicates the deleted region and the resulting deletion junction, and the sequencing chromatograms show the correctly joined upstream and downstream flanking sequences, confirming successful and accurate construction of the seamless Δ*baeS* mutant.

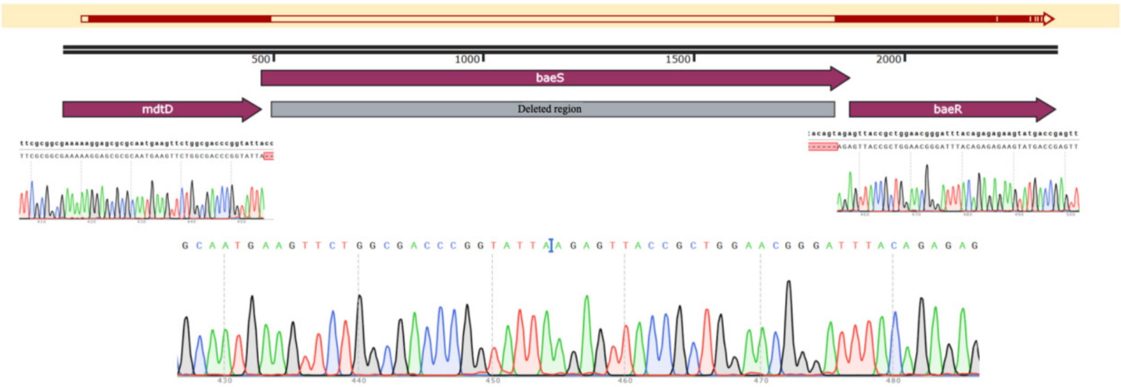

Supplement: Supplementary file 1 [file microorganisms-14-01031-s001.zip › microorganisms-4247954-supplementary.pdf]
